# The effect of berberine and fenugreek seed co-supplementation on inflammatory factor, lipid and glycemic profile in patients with type 2 diabetes mellitus: a double-blind controlled randomized clinical trial

**DOI:** 10.1186/s13098-022-00888-9

**Published:** 2022-08-23

**Authors:** Shima Nematollahi, Gholam Reza Pishdad, Mehrnoosh Zakerkish, Foroogh Namjoyan, Kambiz Ahmadi Angali, Fatemeh Borazjani

**Affiliations:** 1 Nutrition and Metabolic Diseases Research Center and Clinical Sciences Research Institute, Ahvaz Jundishapur University of Medical Sciences, Ahvaz, Iran; 2 Endocrine and Metabolism Research Center, Shiraz University of Medical Sciences, Shiraz, Iran; 3 Health Research Institute, Diabetes Research Center, Ahvaz Jundishapur University of Medical Sciences, Ahvaz, Iran; 4 Research center for Traditional Medicine and History of Medicine, Shiraz University of medical sciences, Shiraz, Iran; 5 Department of Statistics and Epidemiology, School of Health Sciences, Ahvaz Jundishapur University of Medical Sciences, Ahvaz, Iran; 6 Nutrition and Metabolic Disease Research Center and Clinical Sciences Research Institute, Ahvaz Jundishapur University of Medical Science, Ahvaz, Iran

**Keywords:** Berberine, Fenugreek seed, Type 2 diabetes mellitus, Glycemic status, Inflammation, Lipid profile, Persian medicine

## Abstract

**Background:**

Type 2 Diabetes mellitus is one of the most common chronic diseases in the world and has many complications. Due to the importance of using alternative therapies in managing symptoms of this disease, the present study was designed and conducted to investigate the effect of co-supplementation of berberine and fenugreek in patients with type 2 diabetes mellitus.

**Methods:**

A randomized controlled clinical trial was conducted on 50 patients with type 2 diabetes mellitus. Participants were randomized in the intervention group, which received 3 capsules/day of 500 mg (300 mg of berberine + 200 mg of fenugreek seed powder) or placebo for 12 weeks. Biochemical and anthropometric variables were measured at the beginning and end of the study.

**Results:**

We observed that fasting insulin, HbA1C, and hs-CRP significantly decreased in the intervention group compared to the baseline. The mean difference in insulin resistance (-0.32 vs. 0.15), fasting blood sugar (-14.40 vs. 1.68), and fasting insulin (- 2.18 vs. 1.34) were clinically significant in comparison to the control group. Almost all domains of SF-12 scores were significantly higher in the intervention group than in the placebo group.

**Conclusions:**

The combination of berberine and fenugreek seed can improve cardio-metabolic status in patients with diabetes and support the anti-diabetic and anti-inflammatory role of herb in the improvement of quality of life.

**Supplementary Information:**

The online version contains supplementary material available at 10.1186/s13098-022-00888-9.

## Background

Type 2 diabetes mellitus (T2DM), which is on the rise, is one of the most prevalent chronic diseases in both developing and developed nations and is expected to impact more than 693 million individuals by the year 2045 [1, 2]. Patients with T2DM are at risk for developing several acute and chronic complications, including cardiovascular disease (CVD), non-alcoholic fatty liver disease (NAFLD), some types of cancer, and microvascular complications [2]. Insulin resistance (IR) is one of the major T2DM risk factors. [3]. Insulin resistance and hyperglycemia increases the production of free radicals and reactive oxygen species (ROS), which leads to lipid peroxidation and degradation of antioxidant enzymes [4]. Many diabetics have dyslipidemia and are at risk for CVD [5].

Hypertriglyceridemia, hypercholesterolemia, increased levels of low-density lipoprotein (LDL), and decreased levels of high density lipoprotein (HDL) are the characteristics of diabetic dyslipidemia, which appears to be an early event in the development of type 2 diabetes mellitus (T2DM) [6].

Traditional herbal medicines have a long history of use in many countries. Active substances obtained from herbs are now a substantial component of the contemporary pharmaceutical industry  [7–11]. *Trigonellafoenum-graecum*, commonly known as "*Fenugreek seed*", is a popular herb in Africa, India, Iran, South and Central Asia [12]. This herb has various active compounds, including steroids, lipids, alkaloids, saponins, flavonoids, hydrocarbons, galactomannan fiber, and amino acids. Diosgenin is considered the most bioactive compound in fenugreek seed and has strong antioxidant properties. It has been reported that these active ingredients can prevent diabetes by several mechanisms, including β-cell renewal, insulin secretion stimulation, and increased glucose uptake in HepG2 cells with overexpression of the glucose transporter 2 (GLUT-2) [13]. Moreover, diosgenin increases the mRNA expression levels of CCAAT/enhancer-binding protein (C/EBP) and peroxisome proliferator-activated receptor-γ (PPAR-γ) [13, 14].

Berberine is an alkaloid compound with a wide range of therapeutic activities and is derived from the *Hydrastiscanadensis* and *Coptischinensis *[15, 16].

According to experimental investigations, berberine consumption significantly correlated with changes in the gut microbiome, including an increase in good bacterial strains and the production of short-chain fatty acids (SCFA) [17, 18].

Numerous investigations have been undertaken in recent years on the anti-inflammatory, antioxidant, and lipid-lowering benefits of these two substances. Some of these studies have shown encouraging results, while others have yielded insignificant findings [15, 19].

The recent Meta-analysis revealed that berberine consumption is clinically safe and well tolerated by the human body [20], and no adverse effect was observed on participants’ diet [21].

Literature reports berberine's positive effects on managing symptoms of chronic disorders such as glycemic control and improving lipid profiles; however, certain studies were unable to find the strong evidence in this regard [20, 22].

Based on the findings of our investigation, no study has examined the effects of co-supplementing these two herbs on T2DM patients, even though other studies have analyzed the effects of berberine and fenugreek on aspects of glycemic and lipid profiles.

Thus, the goal of the current study was to determine how co-supplementing with berberine and fenugreek seeds affected T2DM patients' glycemic indices, inflammatory marker, and lipid profiles.

## Methods

### Study population

This study is a double-blinded randomized controlled trial conducted on the research and education association of Diabetes, at Shiraz University of Medical Science, Iran, between 2020 and 2021, Shiraz, Iran. Shiraz Diabetes Educational Research Association received referrals from patients diagnosed with T2DM, who were then recruited as study participants. This study enrolled individuals aged between 18–70 years diagnosed with T2DM for at least one year ago; hemoglobin A1c (HbA1c) > 7.0% or fasting blood glucose (FBG) > 7.0 mmol/L; 3) body mass index (BMI) more than 25 kg/m2 and less than 35; 4) no medical history of type 1 diabetes, cardiovascular, thyroid, kidney disease, cancer, mental disorders and taking medications related to the mentioned diseases; 5) and for female patients, a negative pregnancy test. We did not include patients with a BMI > 35, who had been on weight loss diets or taken weight loss supplements for less than 6 months, a history of fenugreek or berberine allergies, and breastfeeding women. None of the patients regularly smoked, drank alcohol, or took psychiatric medications or insulin. Additionally, individuals were excluded if their drug regimen changed throughout the course of the trial, if they started receiving insulin therapy, if they became pregnant, or if they no longer wanted to comply. All study procedures were approved by the Ethics Committee of Ahvaz Jundishapur university of medical sciences (Ethical NO.IR.AJUMS.REC.1398.735) and registered with the Iranian Clinical Trials Registry (IRCT registration number: IRCT20191229045937N1).

### Participant recruitment and screening

Based on the insulin plasma level with a standard deviation of 17.46 in the previous study [23], 19 patients have been estimated for each studied group considering α = 0.05 and a power of 85%. To increase the accuracy and probability of sample loss, 25 patients were considered for each group. All patients signed written informed consent. Current study was designed based on the CONSORT statement for randomized clinical trials [24].

**Fig.1:**
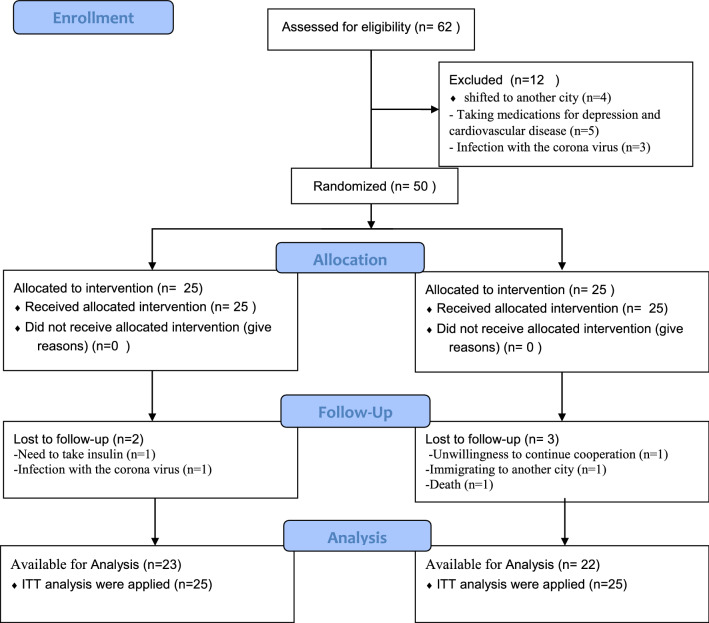
Flow of study

Sixty-two patients with diabetes were initially evaluated, and fifty were eligible to enter the study. Twelve patients were not included in the study due to living in another city, taking medications for depression and cardiovascular disease, as well as coronavirus. Eventually, fifty patients with T2DM were randomly divided into two groups using a block randomization procedure 25 patients in each intervention and placebo groups with three blocks in equal proportions. Randomization was made using computer-generated random numbers by a third person to reduce the bias. Figure 1 shows the flowchart of the participant's enrollment.

The diagnosis of T2DM was performed based on American Diabetes Association guidelines. Patients with fasting blood sugar (FBS) ≥ 126 mg/dl, 2-h plasma glucose ≥ 200 mg/dl, or Glycated hemoglobin ≥ 6.5% were diagnosed with diabetes mellitus [25].

### Study design and interventions

Following the baseline measurements, participants were randomized to receive either 500 mg 3-per-day combination of berberine and fenugreek seed (300 mg of berberine + 200 mg of fenugreek seed powder, Arjuna Natural Pvt. Ltd company, Kerala India) and patients in the placebo group also received 3 capsules containing wheat flour that look similar to the supplements in term of shape, size, and weight. For 12 weeks, participants in both groups were instructed to take the supplements 30 min before the main meals.

Each 500 mg capsule contains 300 mg of berberisaristata extract and 200 mg of fenugreek extract, all of which were standardized to contain 285 mg of berberine hydrochloride NLT and 100 mg of saponins. In the current study, we studied the effect of co-supplementing these herbs in the lower dose, as there have been no side effects documented for any of these herbs at this dose [26], but earlier studies utilized large doses of each of these supplements, and some side effects were observed [26, 27].

The patients and researchers were unaware of the contents of the groups until the study's conclusion. The supplements of the intervention and control groups were coded by a third party (not a relative of the study researcher) using a combination of numeric and alphabetic numbers generated by a computer. To assure the ingestion of the placebo or intervention capsules, calls or texts were made. Each time they came in, patients were requested to bring back their empty supplement boxes so that the remaining ones could be counted if they weren't used. Patients whose compliance was lower than 90% were not included in the trial. The data monitoring committee oversaw the present study's safety and any potential negative effects of the intervention.

### Measurements

Demographic information of the patients was recorded, and their physical activity was assessed by the short version of the international physical activity questionnaire (IPAQ).

Each patient's weight was determined during the first and twelve weeks of the study using Seca scales (Seca Company, Model 769, Germany) with an accuracy of 100 g while wearing light clothing, and their height was determined while wearing no shoes using a wall-mounted meter with an accuracy of 0.5 cm. At the start (week 0) and end of the trial, anthropometric measurements such as height, weight, hip circumference (HC), and waist circumference (WC) were taken for each participant.

BMI was determined by dividing the weight in kilograms by the square of the height in meters2. At the beginning and end of the trial, patients underwent telephone or in-person interviews to complete 3-day dietary recalls covering one holiday and two working days. These questionnaires were analyzed using Nutritionist IV (N4) software designed based on the United States Department of Agriculture Food Composition Table, which was modified for Iranian dietary food items [28].

All patients were asked not to change their routine dietary patterns and physical activity.

The gastrointestinal symptoms such as gastroesophageal reflux, dyspepsia, esophageal motility disorders, gastric motility disorders, and colonic motility disorders were assessed by the questionnaire [29]. The severity of the gastrointestinal symptoms were classified as asymptomatic, sometime, and permanent.

The quality of life (QOL) was assessed and scored by the Persian version of the questionnaire (SF-12) [30]. It includes 12 questions and 8 scales: physical functioning (PF-2 items on limitations doing moderate activities and climbing several flights of stairs), role limitations due to physical problems (RP-2 items on less accomplishment than one would like to achieve, and limitation in kind of work or other activities), bodily pain (BP-1 item on pain interference with one's normal work), general health (GH-1 item on general health perception), vitality (VT-1 item on having energy), social functioning (SF-1 item on the interference of physical health or emotional problems with one's social activities), role limitations due to emotional problems (RE-2 items on less accomplishment than one would like to achieve and not being careful in doing activities as usual) and perceived mental health (MH-2 items on feeling calm or peaceful and feeling sad or blue). Response categories for items vary from 2- to 6-point scales, and raw scores for items ranging from 1 to 6. After recoding raw scores for some items (that are BP, GH, VT, and one item from MH); then the raw scores could be transformed to provide eight scale scores, each ranging from 0 (the worst) to 100 (the best), with higher scores indicating better QOL.

All of the patients had to fast for 12–14 h before baseline and endpoint blood samples were obtained. The serum was immediately stored at −80 °C until analysis after the blood samples were immediately centrifuged for 10 min at a speed of 1500–2000 rpm. An auto-analyzer was used to measure fasting blood sugar (FBS) using the enzymatic method (Pars Azmoon Co., Iran). The Monobind insulin kit was used to perform ELISA measurements on fasting insulin concentrations (Monobind Inc., USA, product code: 5825-300). Ion exchange chromatography was used to assess glycated haemoglobin (Hb) A1C using the colorimetric method in Pars Azmoon Co., Iran. The updated homeostasis model of assessment 2 (HOMA-2) [31].

To calculate insulin resistance (IR), sensitivity (S)%, and beta cell function (BCF)% were available from the University of Oxford at (http://www.dtu.ox.ac.uk/homacalculator/index.php). For measuring lipid profiles, including triglyceride (TG), total cholesterol (TC), and HDL (high-density lipoprotein), we used the enzymatic kits (Pars Azmoon, Iran). LDL was calculated using the Friedewald formula. Enzyme-linked immunosorbent assay kits (Monobind Inc. Iran) were applied to measure high-sensitivity C-reactive protein (hs-CRP).

### Statistical analysis

We applied SPSS software (version 18; SPSS Inc., Chicago, IL) for statistical analysis. The Kolmogorov– Smirnov test was used to determine data normality. Quantitative and qualitative variables were reported in mean (standard deviation) and frequency (percent frequency), respectively. Comparison of the results within groups after the intervention were assessed using paired sample t test. Independent sample t test was used to compare the results between the two groups (placebo and intervention). The mean changes of measured variables before and after the intervention were reported.In addition, analysis of covariance (ANCOVA), which corrects for baseline values, was used to find any differences between the two groups at the end of the study. Covariates included age, disease duration, dietary energy and macronutrient intake (percent), FBS at baseline, HbA1C at baseline, physical activity, W/H ratio, and body mass index. The intention to treat (ITT) approach was applied to all data. ITT approach involves the analysis of all trial participants who were randomized, regardless of dropout. We initially did the per protocol (complete case) sensitivity analysis and ITT analysis. There was no difference between these two analyses. A straightforward imputation strategy, including baseline data imputation, was used to manage the fully lost at random missing data. The baseline values of the outcome variables, FBS and HbA1C, were taken into account in the study. In addition, qualitative variables were compared by a chi-squared test before and after the intervention. P- value less than 0.05 was considered significant in all analyses.

## Results

Forty-five patients completed the study duration (intervention group = 23, placebo group = 22 patients), and 68% of each studied group were female.

Table 1 represents the fundamental traits of patients with type 2 diabetes mellitus. Age, gender, disease duration, education, and PA did not significantly differ between the two study groups at the outset of the investigation. The physical component score (PCS), score changed significantly from baseline to week 12 and was nearly identical in the intervention and control groups. The intervention group, however, showed a tendency to show greater changes in the mental component score (MCS), score from baseline than the placebo group (full table presented as supplementary 1).

**Table 1:** Basic characteristics of patients with type 2 diabetes

Characteristics	Intervention group	Placebo group	P-value^a^
	(n = 25)	(n = 25)	
Duration of disease (years)	9.92 (4.78)	8.96 (4.95)	0.4
Sex, female, n (%)	17 (68)	17 (68)	1
Education, n (%)			
Illiterate and under diploma	10 (40)	11 (44)	0.8
Diploma	8 (32)	7 (28)	
Academic	7 (28)	7 (28)	
PA (MET minutes/week)			
Baseline	1806.02 (2319.45)	1708.58 (4563.89)	0.1
End of trial	1043.98 (1170.32)	641.78 (909.87)	0.07
P-value^b^	0.9	0.8	
Mental component summary			
Baseline	46.29 ± 11.32	53.18 ± 11.37	0.6
End of trial	55.64 ± 7.15	53.26 ± 8.8	0.9
P-value^b^	0.00001	0.4	
Physical component summary			
Baseline	45.68 ± 8.84	44.92 ± 7.96	0.6
End of trial	49.26 ± 6.05	49.28 ± 5.30	0.9
P-value^b^	0.001	0.003	
Patient's medications n (%)			
Metformin	25 (100)	25 (100)	1
Glibenclamide	8 (32)	3 (12)	0.08
Zipmed	9 (36)	10 (40)	0.7
Atrovastatine	11 (44)	13 (52)	0.5
Losartan	9 (36)	9 (36)	1
Aspirin	5 (20)	2 (8)	0.2
Acarboze	5 (20)	3 (12)	0.4
Gloripa	2 (8)	6 (24)	0.1
Diabezid	4 (16)	3 (12)	0.6

*BMI* body mass index, *HC* hip circumference *PA* physical activity, *WC* waist circumference, *MET* metabolic equivalent of task

^*^Mean (SD)

P-value^a^ P values denote significance of between-group difference (P < 0.05, independent samples t-test or U Mann Whitney)

P-value^b^ P values denote significance of within-group changes (P < 0.05, paired samples t-test or Wilcoxon)

Table 2 represent anthropometric measurements of patients. The mean anthropometric parameters at baseline, such as weight, hip circumference, waist circumference (WC), and BMI, did not substantially differ between the two study groups. The mean of WC was considerably lower in both groups at the conclusion of the trial.

**Table 2:** Basic anthropometric indices of patients with type 2 diabetes

Characteristics	Intervention group (n = 25)	Placebo group (n = 25)	P-value^a^
Weight (kg)			
Baseline	71.51 (10.87)	75.06 (11.16)	0.2
End of trial	70.82 (11.52)	74.18 (9.72)	0.1
P-value^b^	0.1	0.1	
WC (cm)			
Baseline	100.08 (9.77)	97.28 (13.89)	0.2
End of trial	99.32 (10.05)	96.32 (13.58)	0.3
P-value^b^	0.03	0.02	
HC (cm)			
Baseline	103.32 (11.12)	103.4 (11.67)	0.9
End of trial	102.78 (11.16)	102.72 (11.61)	0.9
P-value^b^	0.04	0.06	
BMI (kg/m^2^)			
Baseline	27.56 (4.07)	27.62 (3.31)	0.6
End of trial	27.15 (4.17)	27.17 (3.11)	0.6
P-value^b^	0.06	0.03	

*BMI* body mass index, *HC* hip circumference, *PA* physical activity, *WC* waist circumference, *MET* metabolic equivalent of task

^*^Mean (SD)done

P-value^a^ P values denote significance of between-group difference (P < 0.05, independent samples t-test or U Mann Whitney)

P-value^b^ P values denote significance of within-group changes (P < 0.05, paired samples t-test or Wilcoxon)

Table 3 demonstrated that the baseline mean energy intake of the two study groups was significantly different from the baseline macronutrient consumption of the examined individuals with T2DM.

**Table 3:** Energy and macronutrients intake of patients with type 2 diabetes

Variables		Intervention	placebo	P-value^b^	P-value^c^
		(n = 25)	(n = 25)		
Energy (kcal/day)	Baseline	1481.93 (309.95)	2297.39 (1056.02)	0.001	0.009
	End of trial	1602.22 (491.57)	1833.64 (659.62)	0.1	0.5
	P-value^a^	0.2	0.001		
Protein (%)	Baseline	15.07 (2.81)	15.75 (5.58)	0.9	0.8
	End of trial	14.57 (3.14)	15.58 (3.02)	0.2	0.1
	P-value^a^	0.5	0.9		
Carbohydrate (%)	Baseline	53.29 (6.09)	55.75 (9.53)	0.2	0.3
	End of trial	54.53 (8.19)	55.25 (5.62)	0.7	0.7
	P-value^a^	0.4	0.8		
Fat (%)	Baseline	32.90 (5.29)	29.24 (7.00)	0.04	0.1
	End of trial	32.09 (6.91)	30.35 (5.55)	0.3	0.2
	P-value^a^	0.5	0.4		
Vitamin A(RE)	Baseline	2386.78 (3043.75)	1751.15 (1129.17)	0.5	0.4
	End of trial	1052.70 (1175.05)	2238.34 (2154.08)	0.04	0.09
	P-value^a^	0.5	0.2		
Vitamin E (mg)	Baseline	13.96 (13.45)	16.36 (13.16)	0.2	0.2
	End of trial	11.74 (12.99)	18.72 (18.24)	0.2	0.3
	P-value^a^	0.4	0.8		
Vitamin C (mg)	Baseline	99.15 (41.38)	147.94 (63.59)	0.02	0.01
	End of trial	77.13 (23.80)	130.27 (61.25)	0.0001	0.002
	P-value^a^	0.7	0.4		
Vitamin K (mcg)	Baseline	176.16 (81.72)	287.67 (199.55)	0.02	0.01
	End of trial	216.48 (105.84)	331.53 (158.08)	0.03	0.1
	P-value^a^	0.7	0.5		
Potassium (mg)	Baseline	1837.30 (336.40)	2983.61 (2857.04)	0.09	0.06
	End of trial	1794.39 (392.28)	2534.83 (757.47)	0.004	0.005
	P-value^a^	0.004	0.5		
Calcium (mg)	Baseline	493.70 (147.54)	722.60 (389.81)	0.009	0.02
	End of trial	472.19 (231.64)	571.94 (179.66)	0.2	0.4
	P-value^a^	0.9	0.001		
Magnesium (mg)	Baseline	156.57 (27.70)	266.47 (323.68)	0.1	0.1
	End of trial	151.56 (25.94)	211.14 (65.50)	0.0001	0.003
	P-value^a^	0.4	0.3		
Selenium (mg)	Baseline	0.15 (0.43)	0.019 (0.027)	0.3	0.2
	End of trial	0.0041 (0.003)	0.199 (0.635)	0.3	0.7
	P-value^a^	0.3	0.5		

^*^Mean (SD)

P-value^a^ P values denote significance of within-group changes (P < 0.05, paired samples t-test or Wilcoxon)

P-value^b^ P values denote significance of between-group difference (P < 0.05, independent samples t-test or U Mann Whitney)

P-value ^c^ P values denote significance of between-group difference (P < 0.05, ANCOVA) in adjusted model for age, disease duration, PA, and waist to hip ratio

In the intervention group, fasting insulin and HbA1c levels were considerably lower at the end of the trial compared to other observed glycemic indices shown in Table 4. There was no discernible difference between the two groups in terms of fasting insulin at the start and conclusion of the trial, according to the crude and modified models, though.

**Table 4:** Glycemic indices and lipid profile in patients with type 2 diabetes

Glycemic indices and lipid profile		Intervention group (n = 25)	Placebo group (n = 25)	P- value^c^	P- value^d^	P- value^e^
FBS (mg/dl)	Baseline	163.92 (56.27)	137.64 (41.50)	0.03		
	End of trial	149.52 (54.44)	139.32 (26.79)	0.7	0.7	
	P- value^a^	0.1	0.2			
	Mean difference	−14.40 (47.33)	1.68 (36.21)			0.05
	P- value^b^	0.08	0.8			
Fasting insulin (μU/ml)	Baseline	14.36 (10.29)	14.73 (9.52)	0.8		
	End of trial	12.18 (8.66)	16.08 (10.81)	0.1	0.8	
	P- value^a^	0.05	0.3			
	Mean difference	−2.18 (5.42)	1.34 (7.51)			0.1
	P- value^b^	0.05	0.3			
HbA1C (%)	Baseline	8.26 (1.87)	7.12 (1.42)	0.01		
	End of trial	7.72 (1.72)	6.76 (0.90)	0.01	0.02	
	P- value^a^	0.02	0.08			
	Mean difference	−0.54(1.09)	−0.35(0.98)			0.4
	P- value^b^	0.02	0.08			
hs. CRP (mg/ml)	Baseline	3.68 (5.55)	1.13 (1.11)	0.2		
	End of trial	1.6 (1.9)	1.08 (0.85)	0.8	0.6	
	P- value ^a^	0.006	0.4			
	Mean difference	−2.07 (3.92)	−0.22 (0.89)			0.05
	P- value^b^	0.01	0.2			
TG (mg/dl)	Baseline	130.40 (70.02)	122.36 (53.74)	0.8		
	End of trial	135.56 (80.79)	115.24 (36.74)	0.4	0.5	
	P- value^a^	0.1	0.4			
	Mean difference	5.16 (83.40)	−7.12 (51.10)			0.5
	P- value^b^	0.7	0.4			
Total cholesterol (mg/dl)	Baseline	148.04 (28.55)	140.84 (19.91)	0.1		
	End of trial	153.00 (29.32)	150.00 (41.07)	0.9	0.02	
	P- value^a^	0.3	0.08			
	Mean difference	4.96 (23.82)	16.16 (44.69)			0.6
	P- value^b^	0.3	0.08			
HDL-C (mg/dl)	Baseline	38.68 (6.94)	43.24 (9.55)	0.1		
	End of trial	40.46 (7.39)	44.26 (9.04)	0.1	0.02	
	P- value^a^	0.05	0.4			
	Mean difference	1.78 (4.31)	1.02 (6.01)			0.4
	P- value^b^	0.05	0.4			
LDL-C (mg/dl)	Baseline	99.80 (147.03)	66.52 (17.68)	0.1		
	End of trial	71.69 (19.80)	75.83 (30.73)	0.9	0.07	
	P- value^a^	0.3	0.07			0.4
	Mean difference	−28.10 (147.31)	9.31 (24.26)			
	P- value^b^	0.3	0.06			
HOMA2.B	Baseline	57.03 (37.03)	77.18 (50.58)	0.1		
	End of trial	61.04 (39.85)	73.82 (48.30)	0.3	0.6	
	P- value^a^	0.3	0.8			
	Mean difference	4.00 (20.69)	−3.36 (32.23)			0.6
	P- value^b^	0.3	0.6			
HOMA2.IS	Baseline	77.02 (54.75)	78.59 (60.39)	0.9		
	End of trial	85.52 (55.99)	75.86 (62.20)	0.2	0.6	
	P- value^a^	0.1	0.7			
	Mean difference	8.49 (29.75)	−2.72 (53.06)			0.5
	P- value^b^	0.1	0.8			
HOMA2.IR	Baseline	2.08 (1.43)	2.07 (1.27)	0.9		
	End of trial	1.76 (1.19)	2.23 (1.43)	0.2	0.9	
	P- value^a^	0.09	0.4			
	Mean difference	−0.32 (0.87)	0.15 (1.11)			0.07
	P- value^b^	0.07	0.4			

*FBS* fasting blood sugar, *HbA1C* Glycated hemoglobin (Hb) A1C, *hs. CRP* high-sensitivity C-reactive protein, *TG* triglyceride, *HDL-C* high-density lipoprotein cholesterol, *LDL-C* low-density lipoprotein cholesterol, *HOMA B* homeostatic model assessment of β-cell activity, *IS* homeostatic model assessment of insulin sensitivity, *HOMA-IR* homeostatic model assessment of insulin resistance

^*^Mean (SD)

P-value P values denote significance of within-group changes. (P < 0.05, paired-t test (for normal distribution data) and Wilcoxon test (for non normal distribution data

P- value^b^ P values denote significance of within -group mean difference, one sample test was used

P- value^c^ P values denote significance of between-group difference. (P < 0.05, Mann–Whitney *U* test (for fasting blood sugar (FBS)) and independent *t* test (for other variables)

P-value^d^ P values denote significance of between-group difference. (P < 0.05, analysis of covariance (ANCOVA) in the adjusted models (adjusted for age, duration of disease, dietary intake of energy, and macronutrient (%),FBS at baseline, HbA1C at baseline, physical activity, W/H ratio and body mass index))

P-value^e^ P values denote significance of between-group mean difference. (P < 0.05, analysis of covariance (ANCOVA) in the adjusted models (adjusted for age, duration of disease, dietary intake of energy and macronutrient (%), and body mass index))

Table 5 indicates that there is no significant difference between the two groups' baseline recorded gastrointestinal problems. The sole difference between the two study groups at the end of the intervention was esophageal motility problems.

**Table 5:** Severity of the gastrointestinal symptoms before and after intervention with BBR and fenugreek seed

Gastrointestinal symptom	Placebo group (n = 25)			Intervention group (n = 25)			P- value
	Asymptomatic	Sometime	Permanent	Asymptomatic	Sometime	Permanent	
Gastro- esophageal reflux							
Baseline	14 (56)	4 (16)	7 (28)	12 (48)	7 (28)	6 (24)	0.592
End of trial	13 (52)	10 (40)	2(8)	14 (56)	7 (28)	4 (16)	0.540
P- value		0.112			0.392		
Esophageal motility disorders							
Baseline	21 84)	2 (8)	2 (8)	16 (64)	6 (24)	3 (12)	0.237
End of trial	20 (80)	2 (8)	3 (12)	6 (24)	18 (72)	1 (4)	0.0001
P- value		0.607			0.009		
Dyspepsia							
Baseline	10 (40)	14 (56)	1 (4)	6 (24)	14 (56)	5 (20)	0.160
End of trial	8 (32)	12 (48)	5 (20)	8 (32)	9 (36)	8 (32)	0.571
P- value		0.250			0.317		
Gastric motility disorders							
Baseline	8 (32)	10 (40)	7 (28)	6 (24)	11 (44)	8 (32)	0.819
End of trial	8 (32)	11 (44)	6 (24)	7 (28)	10 (40)	8 (32)	0.819
P- value		0.846			0.846		
Colonic motility disorders							
Baseline	19 (76)	5 (20)	1 (4)	12 (48)	7 (28)	6 (24)	0.064
End of trial	19 (76)	5 (20)	1 (4)	13 (52)	8 (32)	4 (16)	0.164
P- value		0.942			0.223		

^*^n (%)

Data are expressed as percent of relative frequency of gastrointestinal symptoms

P < 0.05 considered as significant from chi squared test

## Discussion

There is no evidence to our knowledge on the combined effect of berberine and fenugreek seed on T2DM patients and the majority of the evidence is on the single herb. In the current study, taking three capsules of Berberine and Fenugreek seed per day resulted in a significant reduction in FBS and hs-CRP. Furthermore, only the intervention group's BCF % and IS were higher and clinically significant when compared to the control group. Fasting insulin, IR, and FBS levels were all lower and only clinically significant when compared to the control group.

Besides, most the subscales in SF-12 scores were significantly higher in the intervention group than in the placebo group. These findings support that daily co-supplementation of Berberine and Fenugreek could potentially suppress chronic inflammation, better control disease, and improve health-related quality of life.

The permanent esophageal motility disorders were observed only in one intervention group participant. It should be noted that esophageal motility disorder has not previously been reported as a complication of berberine or fenugreek. Hence, it seems that the combination of the two herbs is more appropriate than their single high-dose consumption. Diabetes is a chronic disease that causes severe complications in other vital organs such as the heart, eyes, and kidneys. It also has a negative impact on the economy by increasing the risk of premature death with disability due to diabetes, as well as absence from work and education [32].

Furthermore, gastrointestinal symptoms are common in patients with diabetes, including constipation, bloating, and gastroesophageal reflux [29].

Our findings showed that fasting insulin and HbA1C were significantly improved after intervention and FBS, BCF%, IS, and IR only clinically improved. In line with our findings, Yine et al. found a significant reduction in the serum concentration of FBS, HbA1C, and postprandial blood glucose after the supplementation with 0.5 g berberine thrice a day for 3 months; however, they reported gastrointestinal discomfort in the intervention group [33].

Another RCT study was conducted in the south-west of Iran showed that 500 mg twice daily berberine supplementation for 4 weeks led to a significant reduction in the FBS, but not fasting insulin, HOMA-β%, and HOMA-I than the control group [34]. A recent meta-analysis of 18 clinical trials showed that berberine supplementation significantly reduced TG, FBS, LDL, increased HDL; reduced insulin resistance to improve type II diabetes, and prevent diabetic encephalopathy [21].

Berberine has been shown in clinical studies to be clinically safe and well tolerated by humans. Few unfavorable side effects have been reported, and no adverse effects on the diet of individuals have been detected. Lee et al. discovered that berberine inhibits fat-forming and lipogenic genes, resulting in less fat formation [35].

Heat generation and oxygen demand are enhanced, and glucose and fat metabolism are accelerated when the expression of uncoupling protein mRNA in skeletal muscles is elevated. This herbal composition also acts as an AMP-activated protein kinase (AMPK) agonist, activating AMPK to boost energy generation and decrease energy storage.Upregulation of AMPK can help to correct lipid, glucose, and energy imbalances, as well as ameliorate metabolic imbalances induced by metabolic diseases [36].

Various mechanisms have also been proposed for the glycemic profile enhancing effects of berberine, such as increased insulin sensitivity, activation of protein kinase (AMPK) 1 by adenosine monophosphate 2 (AMP2), inhibition of gluconogenesis, stimulation of glycogenesis, GLP-13 secretion, and expression of LDL receptor mRNA secretion [37]. By enhancing Acetyl-CoA carboxylase (ACC) phosphorylation, stimulating AMPK can also boost Glucose transporter type 4 (GLUT4) translocation, so indirectly accelerating the uptake of glucose in the blood and free fatty acids to the mitochondria, both of which lead to the decline of glucose and lipids [38]. In our study, we did not find any significant difference between the two groups in terms of lipid profile. Contrary to our study, the results of a meta-analysis study showed that supplementation with berberine caused a significant reduction in the serum levels of TC, LDL, and TG [39]. This impact is hypothesized to be caused by the upregulation of LDL-R on hepatocytes related to the stabilization of LDL-R mRNA and the suppression of PCSK9 transcription due to the accelerated breakdown of hepatocyte nuclear factor 1a (HNF1a) and decreased proprotein convertase subtilisin/kexin type 9 (PCSK9) mRNA expression [40–42]. Fenugreek plant that contains active ingredients such as galactomannan, saponins, trigonelle, and diosgenin and is used as a spice, herb, food, and medicine [43]. The seeds of the fenugreek plant are the most important and well-studied part of the plant [14]. Fenugreek seed (FS) may be advantageous for modifying human plasma glucose and HbA1C levels, according to earlier studies [44]. Rashid et al. in RCT examined the effects of galactomannan derived from fenugreek in newly diagnosed type-2 diabetics, which is consistent with our findings. For 12 weeks, patients took 1 g/day of galactomannan, the monosaccharide component of Fenugreek gum in capsule form, 1 h before meals at a set time. FBS, HbA1c, TC, TG, and LDL all significantly decreased, according to the findings [45]. Additionally, a multicenter, randomised, placebo-controlled, double-blind experiment among T2DM patients revealed that fenugreek seeds at 500 mg bid significantly lowered FBS and post-prandial plasma sugar levels [46].

Similar results were found in a recent RCT that examined the effects of consuming 5 g of fenugreek seed powder three times per day for eight weeks. The results revealed that fenugreek seed powder supplementation significantly decreased the levels of FBS [27]. Fenugreek seeds have been shown to contain 30% soluble and 20% insoluble fibre, which has been shown to slow down the rate at which glucose is absorbed, presumably as a secondary mechanism for the hypoglycemic action, and to stimulate the release of insulin from pancreatic beta cells [47, 48]. In our study, LDL and HDL concentrations improved more than other lipid profiles, however in the adjusted model, TC rose in both groups while rising even more in the control group. In contrast to the findings of our investigation, Ghahdarijani et al. found that fenugreek ingestion significantly reduced TC, LDL, and TG levels while also raising HDL levels [49].

Polyphenols are the most abundant components in this herb that could improve hyperlipidemia. The Hypocholesterolemia effect of Fenugreek was due to the saponin component in it, which efficiently prevents cholesterol synthesis and therefore effectively reduces TC levels [50]. Additionally, fenugreek contains a gel-like dissolvable fibre that, when combined with bile acids, creates excessively large micelles that are difficult for the digestive system to absorb and lower levels of TG and LDL [51]. The presence of 4-hydroxy isoleucine in fenugreek seed also appears to improve the lipid profile and boost insulin release from pancreatic beta cells, which is another way by which it appears to lower blood cholesterol levels [49].

The other beneficial effect of fenugreek is to modulate intestinal microbiota, which can in turn impact metabolic physiology. Studies have shown that the composition of the gut microbiota is impaired in patients with T2DM, and fenugreek supplementation can delay the progression of T2DM and its complications by improving the gut microbiome [52, 53].

In the current investigation, it was determined that an appropriate strategy for the supportive treatment of diabetes was the combination of low-dose berberine and fenugreek seeds, which caused fewer gastrointestinal symptoms than either dose alone.

In a study, Yin et al. evaluated the effects of 500 mg/TDS berberine consumption on the patients with T2DM, and the results showed the occurrence of gastrointestinal symptoms including constipation, flatulence, and abdominal pain [33].

Additionally, fenugreek has been linked to side effects such diarrhoea, indigestion, stomach distention, and bloating when taken orally, according to a study [54].

Therefore, a low-dose mixture of berberine and fenugreek seeds can be used for diabetics as herbal medicines because there were no variations in the occurrence of gastrointestinal symptoms between the two groups before and after the intervention, with the exception of esophageal (Additional file 1) motility abnormalities. Furthermore, one person in the therapeutic group occasionally experienced an esophageal motility issue alone. It is important to note that esophageal motility issue has not yet been listed as a typical side effect of berberine or fenugreek.

Additionally, none of the subjects experienced significant gastrointestinal issues. On the other hand, numerous studies have demonstrated that some herbal substances have the potential to have beneficial effects on other acute or chronic diseases, the risk of which is higher in individuals with diabetes [55, 56].

It goes without saying that diabetes may have an impact on a patient's health and quality of life. Similar to individuals with type 1 diabetes, those with type 2 diabetes have a lower quality of life than those in good health. As a result, several areas, such as physical and psychological health, would be used to evaluate health-related quality of life.

In the current study, the SF-12 subscale in intervention group significantly improved scores for general health (GH), vitality (VT), and mental health (MH), physical functioning(PH),Role physical(RP),social functioning(SF), role emotional(RE),physical component summary (PCS) score and also increased the mental component summary (MCS) score. Similarly, in other clinical studies, intervention with herbal medicine and symbiotic augmented the quality of life scores for some of the subscales [57, 58].

Moreover, findings of a recent Meta-analysis with the inclusion of eighteen studies concluded that physiological or clinical outcomes, and westernized diet were associated with the QOL of type 2 diabetes mellitus patients [59].

The current study assessed for the first time the combined effect of berberine and fenugreek seed on glycemic indices, inflammatory factor, and lipid profiles in T2DM patients.

Contrary to the findings of earlier research, one of the main reasons for the discrepancy between the results of this investigation and the lack of a significant effect after supplementation with berberine and fenugreek on a number of parameters was connected to the dose utilized. As mentioned, we used the lower dose of these two plants with the aim of better gastrointestinal tolerance, and it seems that this dose does not have a significant effect on some biochemical factors. Despite the strengths of this study, however, it suffered from some limitations.

To begin, the current study was conducted during the COVID-19 epidemic. Despite our advice to avoid lifestyle changes during the intervention, particularly physical activity and dietary intake, their PA decreased compared to baseline, which can affect the accuracy of the results. Previous research has also found that PA decreased during the COVID-19 [60]. Second, due to budget constraints, we were unable to measure additional inflammatory factors. Third, because we used exclusion criteria to reduce the effect of confounding variables, we were unable to generalize these findings to all diabetics. Finally, one of the major issues with herbal compounds is their bioavailability.

Despite the fact that these supplements were provided by a pharmaceutical company, no information on the bioavailability of the phytoconstituents was available. Various measures can be taken to improve the rate of clinical translation of potential phytomolecules [61].

The supplements used in this study were formulated in such a way as to have the desired amounts of active compounds, especially saponins, although the bioavailability of these compounds is not yet clear.

## Conclusions

In conclusion, our findings revealed some beneficial effects of berberine and fenugreek seed on glycemic indices, inflammatory factors, and lipid profiles in T2DM patients. Furthermore, there were no significant gastrointestinal side effects with this combination. As a result, consuming a combination of berberine and fenugreek seed appears to be an appropriate strategy for improving diabetes symptoms and quality of life. Further well-designed studies with a longer intervention period, multiple centers, and a larger sample size are suggested without the limitations of our study.

## Supplementary Information

**Additional file 1: Table S1.** Baseline and end of trial scores for the SF-12 domains short-form health survey with berberine and fenugreek seed intervention.

## Data Availability

The datasets used and/or analyzed during the current study are available from the corresponding author on request.

## Abbreviations

ACC: Acetyl-CoA carboxylase
AMP: Adenosine monophosphate
AMPK: Activation of protein kinase
BBR: Berberine
CVD: Cardiovascular disease
ITT: Intention to treat
FI: Fasting insulin
FG: Fenugreek
FBS: Fasting blood sugar
MD: Mean difference
HDL: High-density lipoproteins
HOMA-2: Homeostasis model of assessment
GLUT4: Glucose transporter type 4
IPAQ: International physical activity questionnaire
GLUT-2: Glucose transporter
LDL: Low-density lipoprotein cholesterol
TG: Triglyceride
TC: Total cholesterol T2DM: Type 2 diabetes
VLDL: Very low–density lipoprotein
QOL: Quality of life
CCAAT: Cytosine-cytosine-adenosine-adenosine-thymidine
