# Supplementary material for: The effect of berberine and fenugreek seed co-supplementation on inflammatory factor, lipid and glycemic profile in patients with type 2 diabetes mellitus: a double-blind controlled randomized clinical trial
Source: Diabetol Metab Syndr. 2022 Aug 23;14:120. doi: 10.1186/s13098-022-00888-9 (PMC9395822; doi:10.1186/s13098-022-00888-9)
Supplement: Supplementary file 1 — Additional file 1: Table S1. Baseline and end of trial scores for the SF-12 domains short-form health survey with berberine and fenugreek seed intervention. [file 13098_2022_888_MOESM1_ESM.docx]

**Additional file Table S1.** Baseline and end of trial scores for the SF-12 domains short-form health survey with berberine and fenugreek seed intervention.

| SF-12 domains | **Placebo group**  **(n=25)** | **Intervention group**  **(n=25)** | **P^b^-value** |
| --- | --- | --- | --- |
| Physical functioning  Baseline  End of trial  P^a^- value | 80.00±35.35  85.22 ±22.70  0.3 | 78.00 ±34.09  84.78± 23.52  0.03 | 0.8  0.9 |
| Role physical  Baseline  End of trial  P- value | 70.00± 26.51  84.09± 16.44  0.001 | 69.00± 31.48  88.04± 15.29  0.001 | 0.9  0.4 |
| Bodily pain  Baseline  End of trial  P- value | 84.00 ±23.80  88.63± 16.77  0.1 | 84.00± 21.50  91.30± 12.17  0.08 | 0.8  0.7 |
| General Health  Baseline  End of trial  P- value | 37.00± 20.56  54.54± 16.16  0.002 | 36.00± 24.02  58.69± 14.31  0.00001 | 0.7  0.3 |
| Vitality  Baseline  End of trial  P- value | 78.00± 27.30  76.13± 29.35  0.7 | 54.00± 35.11  69.56± 30.11  0.03 | 0.01  0.3 |
| Social functioning  Baseline  End of trial  P- value | 81.00 ±26.29  81.81± 22.06  0.4 | 68.00 ±30.20  88.04± 14.82  0.001 | 0.09  0.4 |
| Role emotional  Baseline  End of trial  P- value | 79.00 ±31.19  77.84 ±25.56  0.7 | 67.50 ±33.07  85.86± 20.05  0.002 | 0.1  0.3 |
| Mental health  Baseline  End of trial  P- value | 79.00 ±22.45  86.00± 16.77  0.006 | 74.00 ±21.01  90.76 ±10.80  0.001 | 0.3  0.5 |
| Mental component summery  Baseline  End of trial  P- value | 53.18± 11.37  53.26± 8.80  0.4 | 46.29± 11.32  55.64± 7.15  0.00001 | 0.02  0.3 |
| physical component summery  Baseline  End of trial  P- value | 44.92 ±7.96  49.28± 5.30  0.003 | 45.68± 8.84  49.26 ±6.05  0.001 | 0.6  0.9 |

P^a^- value: within groups, P^b^-value: between groups.

Data are expressed as Mean(SD) of quality of life subscales.
